# Supplementary material for: Mapping the evolution of fertility support policies in China: A content and instrumental analysis
Source: PLoS One. 2025 Oct 9;20(10):e0332137. doi: 10.1371/journal.pone.0332137 (PMC12510515; doi:10.1371/journal.pone.0332137)
Supplement: S1 Appendix — (ZIP) [file pone.0332137.s001.zip › S1 Appendix. 226 original policy documents/183-国家发展改革委关于印发全国农村经济社会发展“十一五”规划的通知(FBM-CLI-4-94900).doc]

国家发展改革委关于印发全国农村经济社会发展“十一五”规划的通知

**发布部门：** [国家发展和改革委员会(含原国家发展计划委员会、原国家计划委员会)](https://www.pkulaw.com/law/chl?Aggs.IssueDepartment=60301) [机构沿革](javascript:void(0))

**发文字号：**发改农经〔2007〕1253号

**发布日期：**2007.06.09

**实施日期：**2007.06.09

**时效性：** [现行有效](https://www.pkulaw.com/law/chl?Aggs.TimelinessDic=01)

**效力级别：** [部门工作文件](https://www.pkulaw.com/law/chl?Aggs.EffectivenessDic=XE0303)

**法规类别：** [农村经济体制改革](https://www.pkulaw.com/law/chl?Aggs.Category=02303)

国家发展改革委关于印发全国农村经济社会发展“十一五”规划的通知
（发改农经[2007]1253号）

各省、自治区、直辖市及计划单列市、副省级省会城市、新疆生产建设兵团发展改革委、教育部、财政部、国土资源部、建设部、交通部、水利部、农业部、文化部、环保总局、统计局、林业局、气象局：
　　根据党的十六届五中、六中全会精神和《[中华人民共和国国民经济和社会发展第十一个五年规划纲要](https://www.pkulaw.com/chl/62f50194a99f4ce8bdfb.html)》的总体部署，结合农村经济社会发展实际，我委组织编制了《全国农村经济社会发展“十一五”规划》（以下简称《规划》）。现将《规划》印发给你们，请结合本地区、本部门实际，切实加强对《规划》实施的组织工作，科学安排政府投资，合理引导社会资源，开拓创新，扎实工作，加快农村经济社会发展，确保顺利实现全国农村经济社会发展“十一五”规划目标。
　　附：全国农村经济社会发展“十一五”规划

中华人民共和国国家发展和改革委员会
二00七年六月九日

　　全国农村经济社会发展“十一五”规划

国家发展和改革委员会
二00七年六月

　　目录

　　一、规划背景
　　(一)“十五”农村经济社会发展成就
　　1、农业综合生产能力稳步提高
　　2、农业和农村经济结构调整成效显著
　　3、农民收入快速增长
　　4、生态环境建设取得重大进展
　　5、农村生产生活条件得到改善
　　6、农村社会事业取得新进展
　　7、农村改革开放迈出重要步伐
　　(二)“十一五”农村经济社会发展环境
　　1、农村经济社会发展出现新变化
　　2、加快农村经济社会发展的有利条件
　　3、农村经济社会发展面临的主要困难
　　二、指导思想和发展目标
　　(一)指导思想
　　(二)发展目标
　　三、主要任务
　　(一)积极发展现代农业
　　1、稳定发展粮食生产
　　2、加快农业科技进步
　　3、健全农业服务体系
　　4、大力发展节约型农业
　　5、加快发展农业循环经济
　　6、提高农业装备水平
　　(二)调整优化农业结构
　　1、积极发展优质农产品
　　2、建设优势农产品产业带
　　3、大力发展畜牧业
　　4、积极发展水产业
　　5、壮大林业产业体系
　　(三)增加农民收入
　　1、挖掘农业内部增收潜力
　　2、大力推进产业化经营
　　3、加快发展农村二、三产业
　　4、发展壮大县域经济
　　5、大力发展劳务经济
　　6、继续做好扶贫工作
　　(四)加强生态环境建设
　　1、加强生态保护
　　2、强化综合治理
　　3、抓好重点工程
　　(五)改善农村生产生活条件
　　1、加强农业和农村基础设施建设
　　2、搞好村镇规划和建设
　　(六)大力发展农村社会事业
　　1、优先发展农村教育
　　2、加强农村公共卫生和基本医疗
　　3、加快农村公共文化建设
　　4、稳定农村低生育水平
　　5、加快建立农村社会保障制度
　　6、加强农村民主政治建设和精神文明建设
　　四、保障措施
　　(一)切实保护好水土资源
　　(二)加强对农业和农村的支持与保护
　　(三)深化农村改革开放
　　(四)大力发展农民专业合作经济组织
　　(五)加强和改善对农村经济的宏观调控
　　(六)增强应对农村重大突发事件的应急反应能力
　　(七)保障农村社会事业健康发展
　　(八)加强农村法制建设

　　全国农村经济社会发展“十一五”规划

　　“十一五”时期是建设社会主义新农村，全面建设小康社会的关键时期。建设社会主义新农村，首要任务是发展农村经济，全面建设小康社会，重点和难点在农村。加快农村经济社会发展，对于建设社会主义新农村，保障国民经济平稳较快发展，推进全面建设小康社会进程，构建社会主义和谐社会具有十分重要的意义。根据党的十六届五中、六中全会精神和《[中华人民共和国国民经济和社会发展第十一个五年规划纲要](https://www.pkulaw.com/chl/62f50194a99f4ce8bdfb.html)》等关于建设社会主义新农村、构建社会主义和谐社会的总体部署和要求，结合农村经济社会发展实际，编制本规划。

一、规划背景

(一)“十五”农村经济社会发展成就
　　“十五”以来，党中央、国务院坚持以科学发展观统领经济社会发展全局，按照统筹城乡发展的要求，适应农业发展进入新阶段和我国加入世界贸易组织后的新形势，实行“多予、少取、放活”的方针，制定出台了一系列支农惠农政策，加大了对农业和农村工作的支持力度，五年中央财政用于“三农”的各项支出达到11300多亿元，加上地方各级政府的支农投入，调动和引导了广大农民发展生产的积极性，农村经济社会发展取得新成就。
　　1、农业综合生产能力稳步提高。粮食生产出现重要转机，2004年以来，粮食播种面积不断扩大，单产连创历史新高，总产连续增加，扭转了2000年以后粮食产量大幅下滑的局面。2005年，全国粮食、棉花、油料、糖料、肉类和水产品产量分别达到48402万吨、571万吨、3077万吨、9452万吨、7743万吨和5108万吨，分别比2000年增长了4．7％、29％、4．1％、23．8％、26．4％和l9．4％。农林牧渔业增加值达到23071亿元，总产值达到39451亿元，按可比价计算，分别比2000年增长21．3％和29．3％。
　　2、农业和农村经济结构调整成效显著。优质农产品得到较快发展，优质专用小麦、优质水稻和“双低”油菜种植面积比例分别达到49％、65％和77％；高效经济作物快速发展，经济作物约占农作物种植面积的33％，比2000年提高了2．3个百分点；农业区域化布局取得重要进展，主要农产品生产迸一步向优势产区集中；畜牧业保持快速增长势头，产值年均实际增长7％，总产值达到13311亿元；农业内部结构和农村产业结构不断优化，农林牧渔业增加值比例由2000年的59．5：4．5：24．9：11．1，调整为2005年的56．6：4．3：28．8：10．3，农业比重下降了2．9个百分点，牧业比重上升了3．9个百分点。第一产业增加值占国内生产总值的比重降到12．6％，第一产业从业人员占全社会从业人员的比重降到44．8％，分别比2000年下降2．2个百分点和5．2个百分点。五年转移农业劳动力4000多万人，扭转了“九五”期间农业从业人员不降反升的局面。
　　3、农民收入快速增长。在农产品价格上涨、政府补贴增加和外出务工环境改善等因素作用下，2005年农民人均纯收入达到3255元，比2000年增加1002元，扣除价格因素，“十五”年均增长5．3％，增速创“七五”以来新高，实现了“十五”计划5％的预期目标。工资性收入成为农民收入的重要来源，2005年农民人均工资性收入达到1175元，占当年农民人均纯收入的36％和“十五”期间农民人均纯收入增量的47％。扶贫开发取得新进展，农村贫困人口减少到2365万人。
　　4、生态环境建设取得重大进展。天然林资源保护、退耕还林、京津风沙源治理、天然草原退牧还草、水土流失综合治理等重点生态建设工程稳步推进，五年造林3933万公顷，治理水土流失面积2400万公顷，治理“三化”草地850万公顷。全国森林覆盖率由16．55％提高到18．21％，荒漠化土地面积和沙化土地面积年均分别减少75．85万公顷和1．83万公顷。全国生态环境总体恶化的势头得到初步遏制，一些重点治理区域生态环境明显好转。
　　5、农村生产生活条件得到改善。南水北调工程开工建设，新一轮治淮工程全面展开。新修江河堤防8600多公里，加固长江干堤3576公里，除险加固病险水库1800余座。大型、中型灌区续建配套和节水改造工程进展顺利，大型粮棉油生产基地、优质粮食产业工程、农业综合开发、动物防疫体系、种养业良种工程等重点农业建设工程稳步推进。农村“六小工程”建设全面展开。五年改造中低产田873万公顷，新增有效灌溉面积l55万公顷，新增工程节水灌溉面积495万公顷，灌溉用水有效利用系数提高到0．45。解决了6700多万农村人口的饮水困难和饮水不安全问题，1800万户农民用上了沼气。全国绝大部分乡镇和建制村已通了公路，约80％的乡镇和60％的建制村通了沥青(水泥)路。农村电网建设与改造全面展开，农村供电质量普遍改善，到户电价明显降低。气象为农业服务水平及防灾减灾能力得到提高。
　　6、农村社会事业取得新进展。基本普及了农村九年义务教育，国家实施“两免一补”政策为中西部地区3400万名贫困家庭学生提供免费教科书。671个县(市)开展了新型农村合作医疗制度改革试点，1．77亿农民参加了合作医疗。新建和改建县级图书馆、文化馆1156个，基本实现了县县有图书馆、文化馆。50户以上已通电自然村基本实现广播电视村村通，解决了近亿农民收听收看广播电视问题。为中西部地区632个低收入县配置了流动电影放映车和放映设备。农村部分计划生育家庭奖励制度已覆盖全国大部分地区，受益人群超过l35万。全国共有1534个县开始试行农村低保制度，享受人数达到777万人。5400多万农民参加了农村社会养老保险。
　　7、农村改革开放迈出重要步伐。农村税费改革全面展开，各地取消了“三提五统”等专门面向农民的收费，减免农业税，取消了除烟叶以外的农业特产税及牧业税，与税费改革前相比，减轻农民税费负担l200多亿元。实行了对种粮农民直接补贴、良种补贴和农机具购置补贴，以及重点短缺粮食品种最低收购价政策，加强了对农业和农民的支持与保护。粮棉流通体制改革进一步深化，农村金融改革步伐加快，土地征用制度改革稳步推进，城乡劳动力市场的制度性分割局面开始转变，农民外出务工环境明显改善。农业对外开放取得新进展，国际合作领域不断扩大，农产品贸易大幅增长。

| 专栏1　“十五”计划农村经济社会发展主要指标完成情况 | | | | |
| --- | --- | --- | --- | --- |
| 指　　标 | 2000年 | “十五” 计划目标 | 2005年 | “十五”年 均增长(％) |
| 耕地保有量(亿公顷) 畜牧业产值占农业总产值的比重(％) 第一产业增加值占国内生产总值的比重(％) 第一产业从业人员占全社会从业人员的比重(％) 五年转移农业劳动力(万人) 农村居民人均纯收入年均增长(％) 森林覆盖率(％) 五年新增治理水土流失面积(万公顷) 五年新增治理“三化”草地面积(万公顷) 灌溉用水有效利用系数 | 1.2827 29.7 14.8 50 16.55 0.42 | 1.28 33 13 44 [4000] 5 18.2 [2500] [1650] 0.45 | 1.22 33.7 12.6 44.8 18.21 0.45 | -1 [4] [-2.2] [-5.2] [4000] 5.3 [1.66] [2400] [850] [0.03] |
| 注：带[ ]的为五年累计数 | | | | |

　　(二)“十一五”农村经济社会发展环境
　　1、农村经济社会发展出现新变化。一是农产品供求关系发生根本性变化，由长期短缺变为总量基本平衡、丰年有余；二是科学发展观和统筹城乡发展战略思想的提出，国家对“三农”工作支持的力度不断加大；三是农村富余劳动力流动规模越来越大，外出打工成为农民增收的重要来源；四是农产品贸易发生重大变化，我国从农产品净出口国转为净进口国；五是农村社会公共服务需求不断增长，社会事业得到高度重视。这些新交化既为农村经济社会发展带来机遇，也带来挑战。
　　2、加快农村经济社会发展的有利条件。根据新的形势，中央提出了坚持科学发展观、统筹城乡经济社会发展、建设社会主义新农村、构建社会主义和谐社会等重大战略思想，为发展农村经济指明了方向；我国总体上已进入了以工促农、以城带乡的发展阶段，国家初步具备了进一步加强扶持“三农”的能力和条件，支农投入不断加大；国民经济发展处于重要战略机遇期，工业化和城镇化快速推进，有利于加快农业劳动力向非农产业和城镇转移；城乡人民生活水平不断提高，对农产品的需求和农业的功能提出了新的要求，有利于拓展农业发展空间；以生物技术、信息技术为核心的农业高新技术快速发展，为缓解农业资源约束，发展现代农业展现了新的前景；随着政府职能不断转变和公共财政不断完善，为改善农村居民教育、卫生、文化生活等条件，促进基本公共服务均等化，提供了强有力的支撑条件；农村综合改革的不断深化和对外开放的不断扩大，为发展农村经济带来新的活力；亿万农民迫切要求改变生产生活现状的强烈愿望和全社会正在形成的关心农业、关爱农民、关注农村的良好氛围，为发展农村经济，建设社会主义新农村营造了广泛的群众基础和良好的社会条件。
　　3、农村经济社会发展面临的主要困难。农业基础设施薄弱，生产力水平较低，抵御自然灾害能力不强，农业总体上仍然是靠天吃饭；耕地减少、淡水紧缺和人口增加的趋势短期内难以改变，生态环境总体恶化的趋势尚未从根本上扭转，资源和环境对农村经济发展的制约日益突出；植物病虫害、动物疫病、农业污染及渔业水域环境恶化等对农业生产及农产品质量安全构成新的威胁；农业生产规模小，农业产业化水平低，农业科技水平不高，不利于提高农业的竞争力和综合效益；农业劳动力素质总体偏低，农村组织化程度不高，与发展现代农业、建设社会主义新农村的时代要求还不能完全适应；农业建设资金缺口较大，资金供求矛盾尖锐，与发展农村生产力，加强农村基础设施建设，提高农村公共服务水平的要求不相适应；农业在经济全球化和区域经济一体化进程中面临的压力增大，农产品国际贸易竞争对国内农业发展带来新的挑战；城乡二元结构仍然比较明显，农村社会事业发展严重滞后，统筹城乡发展的体制和政策尚待建立和完善；深化农村改革、巩固农村税费改革成果的任务仍十分艰巨，促进农业不断增效、农村加快发展、农民持续增收的长效机制尚未建立起来。

二、指导思想和发展目标

(一)指导思想
　　以邓小平理论和“三个代表”重要思想为指导，认真贯彻落实科学发展观，按照建设社会主义新农村和构建社会主义和谐社会的总要求，坚持统筹城乡经济社会发展的基本方略，坚持工业反哺农业、城市支持农村和“多予、少取、放活”的方针，以提高农业综合生产能力为基础，加快科技进步，提高农民素质，积极发展现代农业，转变农业增长方式；以增加农民收入为核心，大力发展二、三产业，加快转移农村富余劳动力，深化改革开放，调动亿万农民的积极性；以加强国家对“三农”的支持和保护为保障，加强农村基础设施建设，加快发展农村社会事业，提高公共服务水平，推动农村经济社会又好又快发展。
　　(二)发展目标
　　按照“生产发展、生活宽裕、乡风文明、村容整洁、管理民主”的总要求，“十一五”时期农村经济社会要努力实现以下主要发展目标：
　　--主要农产品生产能力稳步提高。耕地面积稳定在1．2亿公顷以上，基本农田面积保持在现有水平，粮食综合生产能力达到5亿吨以上，确保国家粮食安全。棉花、油料、糖料、肉类、水产品等主要农产品产量平稳增长。农产品质量安全水平明显改善。动植物保护能力明显加强。
　　--农村经济结构进一步优化。农业内部结构更加合理，畜牧业产值占农业总产值的比重达到38％。农产品区域布局更加合理，优势农产品产业带初步形成。农村产业结构更加协调，二、三产业得到快速发展，食品工业产值与农业产值之比达到0．8：1。农民就业结构逐步优化，五年转移农村劳动力4500万人，全国城镇化率达到47％。
　　--转变农业增长方式迈出重要步伐。农科技创新能力稳步提高，科技对农业增长的贡献率提高约5个百分点。灌溉用水有效利用系数提高到0．5。化肥、农药利用效率提高到35％。农机装备总动力达到8亿千瓦，耕种收综合机械化水平达到45％。
　　--生态环境总体恶化趋势基本遏制。植树造林2000万公顷，森林覆盖率达到20％。新增治理水土流失面积2500万公顷，新增治理“三化”草地5200万公顷，新增治理沙化土地750万公顷，实施生态修复面积3000万公顷。加强农村面源污染防治工作。主要江河湖库二级水功能区水质达标率达到55％。加强自然保护区建设，力争90％的国家重点保护野生动植物物种、50％的自然湿地和70％的重要湿地得到有效保护。
　　--农村生产生活条件得到明显改善。农田有效灌溉面积达到5867万公顷，粮食主产区排涝标准达到3-5年一遇。解决1．6亿农村人口饮水安全问题，农村集中式供水受益人口比例达到55％。适宜农户沼气普及率达到28．8％。新建改建农村公路120万公里。解决农村无电人口基本用电问题。基本实现村村通电话，乡乡能上网。积极治理农村垃圾、污水，农民居住环境明显改善。
　　--农村社会事业得到快速发展。普及和巩固农村义务教育，农村教育条件明显改善。新型农村合作医疗制度基本覆盖农村居民，形成较为完善的县、乡、村三级农村卫生服务网络。基本实现乡镇有综合文化站、行政村有文化活动室。人口稳定在低生育水平，人口素质和结构得到改善。全面建立农村最低生活保障制度，完善农村社会救助体系，社会保障水平明显提高。
　　--农民收入持续增加。按2005年价格计算，农民人均纯收入达到4150元以上，年均增长5％以上，其中工资性收入占农民人均纯收入的比重达到42％以上。农村贫困人口数量进一步减少。
　　--农村和谐社会建设取得重要进展。按照民主法制、公平正义、诚信友爱、充满活力、安定有序、人与自然和谐相处的总要求，以广大农民最关心、最直接、最现实的利益问题为重点，加快改善农村贫困人口生产生活条件，重视和关心农村弱势群体，落实水库移民扶持政策，关爱农村留守儿童和老人生活。加强农村民主法制建设，拓宽农村社情民意表达渠道，建立矛盾纠纷排查调处机制，搞好农村社会治安综合治理，促进农村社会公平正义。培育文明、健康、高尚的思想氛围和道德风尚，努力形成团结、友爱、安定、有序的和谐环境。

| 专栏2　“十一五”时期农村经济社会发展的主要指标 | | | | | |
| --- | --- | --- | --- | --- | --- |
| 类别 | 指　　标 | 2005年 | 2010年 | 年均增长 　(％) | 属性 |
| 优化 农业 结构 | 耕地面积(亿公顷) 粮食综合生产能力(亿吨) 畜牧业产值占农业总产值的比重(％) 食品工业产值与农业产值之比 | 1.22 4.84 33.7 0.5：1 | 1.2 5 38 0.8：1 | -0．3 0.65 [4.3] | 约束性 约束性 预期性 预期性 |
| 转变 增长 方式 | 科技对农业增长的贡献率(％) 灌溉用水有效利用系数 化肥利用效率(％) 农药利用效率(％) 耕种收综合机械化率(％) | 48 0.45 30 30 36 | 53 0.5 35 35 45 | [5] [0.05] [5] [5] [9] | 预期性 预期性 预期性 预期性 预期性 |
| 改善 生态 环境 | 森林覆盖率(％) 造林面积(万公顷) 新增治理水土流失面积(万公顷) 新增治理“三化”草地面积(万公顷) 新增治理沙化土地面积(万公顷) 主要江河湖库二级水功能区水质达标率(％) | 18.21 48 | 20 551 | [1.79] [2000] [2500] [5200] [750] [7] | 约束性 预期性 预期性 预期性 预期性 预期性 |
| 基础 设施 建设 | 农田有效灌溉面积(万公顷) 解决农村饮水安全问题人口(亿人) 发展农村沼气(万户) 新改建农村公路(万公里) | 5667 1800 | 5867 4000 | [200] [1.6] [2200] [120] | 预期性 约束性 预期性 预期性 |
| 提高 生活 水平 | 五年转移农村劳动力(万人) 农村居民人均纯收入(元) 工资性入占农民人均纯收入的比例(％) 农村集中式供水受益人口比例(％) 新型农村合作医疗覆盖率(％) | 3255 36 40 23.5 | ＞4150 ＞42 55 ＞80 | [4500] ＞5 ＞[6] [15] ＞[56.5] | 预期性 预期性 预期性 预期性 约束性 |
| 注：带[ ]的为五年累计数，农民收入按2005年价格计算 | | | | | |

三、主要任务

(一)积极发展现代农业
　　1、稳定发展粮食生产
　　稳定粮食播种面积。坚持最严格的耕地保护制度，强化和落实耕地保护责任制，切实控制农用地转为建设用地的规模。加大土地复垦、整理力度，合理开发后备耕地资源，继续实施东中部粮食主产区基本农田整理工程和西部生态建设地区农田整治工程。加强基本农田保护示范区建设，切实保护好基本农田。提高农作物复种指数。到2010年粮食播种面积稳定在1．053亿公顷以上，其中水稻播种面积稳定在3000万公顷左右。
　　加强耕地质量建设。加强农田水利基础设施建设，加快改造中低产田，改良盐碱地，按照田地平整、土壤肥沃、路渠配套的要求，建设旱涝保收、高产稳产标准农田。加大土壤肥力调查和监测力度，完善全国耕地质量动态监测和预警系统，为农民科学施肥提供指导和服务。实施新一轮沃土工程，引导农民科学施肥，鼓励农民多积农家肥，多施有机肥，增加土壤有机质。推广测土配方施肥，推行有机肥综合利用与无害化处理技术。改进耕作方法，发展保护性耕作。科学使用化肥、农药和农膜，防止化肥、农药、农膜不合理使用对土壤的污染。开展土壤污染现状质量调查，建立土壤环境质量评价和监测制度。结合重大农业项目，加强农村田间机耕道路建设，切实解决农业机械下地难问题。
　　提高粮食单产水平。以四大粮食作物为重点，加快新品种选育，集成推广高效栽培技术和栽培模式，加强农作物病虫害防控，促进良种良法配套。组织实施粮食丰产科技工程和农业生物灾害防控行动，搞好超级稻等粮食新品种的推广工作，主要粮食作物良种普及率稳定在95％以上，病虫害产量损失率控制在5％以下。力争全国粮食单产水平提高到每亩325公斤左右。
　　加强粮食主产区生产能力建设。继续实施大型商品粮生产基地和优质粮食产业工程，在13个粮食主产省和西部重要产粮区，加强小型农田水利、良种繁育、病虫害防治、农机装备推进等基础设施建设，集中连片建设高产稳产标准粮田。实施农业综合开发项目，以粮食主产区为重点，通过水土田林路综合治理，大力改造中低产田，建设旱涝保收、高产稳产高标准农田。支持西部有条件的地区建设区域性商品粮基地，解决西部地区粮食供给问题，促进粮食区域平衡。
　　2、加快农业科技进步
　　建设农业科技创新体系。深化农业科研体制改革，充分发挥农业科研院所、大专院校、涉农企业的作用，优化整合农业科研资源，建设国家农业科技创新体系。加强农业知识产权保护，改善农业技术创新投资环境，鼓励企业建立农业科技研发基地。
　　加强重大农业技术攻关。依靠生物技术和信息技术，加强主要农作物育种和高效栽培、畜牧水产育种和疫病控制关键技术攻关，力争在农业新品种选育、农作物高效栽培模式、农作物病虫害防治和动植物疫病防控、农业资源高效利用、主要生产环节农机装备等方面有新的突破。
　　加快推广农业科技成果。加大农业技术推广力度，实施农业科技入户工程，大力培训科技示范户，积极推广农作物优良新品种和保护性耕作、平衡施肥、水产养殖水质调控、病虫害生物防治等先进实用技术。鼓励农业科技人员采取科技承包等方式，向农民传授先进生产技术。
　　3、健全农业服务体系
　　健全农业良种体系。实施农作物种子工程和畜禽水产良种工程，加强种质资源保护、品种改良、良种繁育、种子质量检测等基础设施建设。改善种质资源保护及品种性能测定设施。推进种子管理体制改革。
　　健全农业技术推广服务体系。按照强化公益性职能、放活经营性服务的要求，推进农业技术推广体系的改革和建设。加强县及县以下推广服务网络建设，强化县乡农技推广机构的服务功能。整合基层涉农服务机构和资源，鼓励建立综合性农业服务站(所)。积极鼓励和支持农业科研单位、涉农企业、农业产业化经营组织、农民专业合作组织等参与农业技术推广服务．规范各类经营性服务组织的行为。健全以农机合作经济组织和农机协会等专业服务组织为龙头、农机大户为主体、农机户为基础的农机社会化服务体系。
　　健全动植物保护体系。加快实施《全国动物防疫体系建设规划》，重点建设动物疫病监测预警、预防控制、防疫检疫监督、兽药质量监察及残留监控体系，稳定乡镇动物防疫机构和队伍，建立健全动物标识和动物疫病追溯体系。完善重大农作物有害生物预警与监控体系，提高植物保护水平。
　　健全农产品质量安全体系。认真贯彻落实《[农产品质量安全法](https://www.pkulaw.com/chl/b6f5f01601279950bdfb.html)》，加强农产品质量安全监督管理，强化农产品生产过程的农药残留监控，提高农产品质量安全监管能力。完善主要农产品质量安全标准、检验方法标准和农业投入品技术标准，加快建设农产品质量安全检验检测体系。在重点地区、品种、环节和企业，加快推行标准化生产和管理。实行添加剂规范使用制度。严格执行转基因食品、液态奶等农产品标识制度。推行地理标志制度和农产品质量安全追溯制度。完善农产品质量安全例行监测制度和信息发布制度。
　　健全农业信息服务体系。整合涉农信息资源，抓好“金农”工程，建设农业监测预警、主要农产品和农业生产资料市场监管、农村市场与科技信息服务等应用系统，初步形成延伸到基层农村的信息网络。结合“宏观经济管理信息系统”(“金宏”工程)建设，继续选择一些农业和农村经济比重大、区域特色明显的市、县建设农村经济动态监测点，提高农村经济信息传递的时效性和准确性。加快建设国家粮食安全预警系统。加强农业龙头企业信息化建设，提高农业企业信息化水平。完善全国水土流失监测系统和管理信息系统。
　　健全农业防灾减灾体系。健全水文测报、洪水预警和防汛抗旱指挥系统。完善重大生物灾害监测预警和应急防控体系与机制。建立森林(草原)火灾监控和防火指挥系统。完善农村气象服务系统，增强农村气象防灾减灾能力。
　　4、大力发展节约型农业
　　加快发展节水灌溉。根据各地水资源承载能力，科学确定农业发展模式、发展规模和农作物结构，在西北等水资源短缺地区和生态脆弱地区，控制发展高耗水农作物。加大灌区续建配套与节水改造力度，采取渠道防渗、管道输水、末级渠系改造、灌区用水计量等措施，减少输水损失，提高渠系水利用率。新建灌区要全部建成节水型灌区。加快推广节水设备和技术，制定优惠政策，鼓励农民购买节水灌溉设备，有条件的地区要积极推广微灌、喷灌、渗灌和滴灌等先进节水技术。干旱半干旱地区要结合解决人畜饮水，积极开展雨水集蓄利用。大力发展旱作节水农业，建设旱作节水农业示范区，加快形成具有区域特色的旱作节水农业发展模式。推进节水增效灌溉示范项目建设。实现全国灌溉用水总量零增长。
　　深入开展节约用地、科学用种、科学施肥、科学用药及节约能源活动。提倡精耕细作，发展间作套种，推广立体种植和立体养殖，提高土地、水面利用率。推广精量半精量播种、种子包衣等技术，提高种子成苗率，节约用种。普及科学施肥知识，推广科学施肥技术，开展测土配方施肥，推广化肥深施，提高肥料利用率。坚持生物防治与药物防治相结合，推广先进适用的施药机械和施药技术，加强安全用药宣传，引导农民按照技术规范科学用药。鼓励生产、使用节电、节油农业机械和农产品加工设备，推广节柴灶具，节约农村能源。
　　5、加快发展农业循环经济
　　积极推广农业废弃物综合利用技术、相关产业链接技术和风能、太阳能、生物质能等可再生能源开发利用技术。大力推广以沼气为纽带、养殖业与种植业相互促进的农业循环经济发展模式，实现人畜粪便和农村有机生活垃圾的无害化处理和资源化利用。提倡秸秆过腹还田，加快秸秆气化技术的试点、总结和推广工作，提高农作物秸秆的利用价值。在坚持以“非粮”作物为主，发展生物质能源、生物化工的同时，加大纤维素乙醇开发力度，促进纤维素乙醇的产业化发展。推广农业清洁生产技术和生产模式，加强农业面源污染防治和水污染防治，促进农业资源循环利用。加强对乡镇企业污染物排放的在线监控，对超标排污企业实行限期治理或取缔。加强农村工业园区“三废”处理设施建设。
　　6、提高农业装备水平
　　大力发展新型农用工业。加强先进适用农机具的研制、示范和推广，力争玉米机械收获取得重大突破，水稻机械化稳步推进，特色农产品机械稳步发展，逐步提高重要农时、重点作物、关键生产环节和粮食主产区机械化作业水平。继续实行农机具购置补贴政策，鼓励和引导农民、农场职工、农机服务组织购买和使用农业机械。完善农机作业市场信息，进一步推进农机跨区作业。加强农机安全生产监管工作，确保农业机械安全运转，高效使用。积极发展高浓度肥料、缓释肥料和复混肥料，加强安全、高效、低毒、低残留农药和可降解农膜的研发及其生产。

专栏3　现代农业建设重点工程 　　大型商品粮生产基地→在粮食主产省及非主产省的重要粮食产区，以地市为单位，集中连片 建设高产稳产大型商品粮生产基地，重点加强小型农田水利、良种繁育等粮食生产基础设施建 设，适当完善病虫害防治等农技服务设施。 　　优质粮食产业工程→在13个粮食主产区的484个粮食主产县(场)，建设标准粮田、良种繁 育、病虫害防控和农机装备推进等项目。 　　农业综合开发→以粮食主产区中低产田改造为重点，综合运用工程、生物和科技等措施，实 行水土田林路综合治理，着力加强农业基础设施，改善农业生产条件，提高农业综合生产能力。 　　现代农业示范建设→以调整农业结构和促进农民增收为目标，继续在全国各地选建一批各具 特色、类型多样的现代农业示范项目，为各地探索现代农业发展新路子发挥引导、示范和辐射带 动作用。 　　基本农田保护示范区建设→以基本农田标准化、基础工作规范化、保护责任社会化、监督管 理信息化为目标，在全国建设116个国家基本农田保护示范区，为探索建立基本农田保护有效机 制发挥示范作用。 　　沃土工程→加强中产田耕地质量建设和土肥新技术创新，完善耕质量监测网络体系，使项目 区的中产田耕地基础地力提高一个等级。 　　种养业良种工程→加强农作物种质资源保存、品种改良、良种繁育及种子质量检测等基础设 施建设，使良种覆盖率稳定在95％左右。建设畜禽水产原、良种场，改善种质资源保护及品种性 能测定设施。 　　动物防疫体系→建设和完善动物疫病监测预警、预防控制、检疫监督、兽药质量监察及残留 监控、防疫技术支撑、防疫物质保障六大系统。重点完善国家、省、县、乡镇四级动物防疫基础 设施，在全国有畜产品产出的2743个县(市)、团场建设动物防疫站，全国2861个县级行政区划单 位建设动物卫生监督站，重点乡镇建设兽医站。 　　植保工程→加强农业有害生物预警与监控体系、优势农产品有害生物非疫区、技术支撑等建 设，提高我国抵御农业有害生物灾害的能力。 　　旱作节水农业示范→根据不同地区地貌、土壤、作物和耕作制度等不同条件和特点，通过工 程、设施、农艺、农机、化学和管理等措施的综合应用，提高田间蓄水、抗旱保水、节水灌溉等 综合节水能力，形成具有区域特色的旱作节水农业发展模式，推进节约型农业建设。 　　农产品质量安全检验检测体系→加强国家、省、县三级农产品质量检测能力建设，初步建立 我国农产品质量安全检验检测体系框架。 　　农村经济信息服务系统→整合涉农信息资源，抓好“金农”工程，建设农业监测预警、主要 农产品和农业生产资料市场监管、农村市场与科技信息服务等应用系统。结合“宏观经济管理信 息系统”(“金宏”工程)建设，加强农村经济动态监测点建设，完善农村经济信息服务体系。 　　农村气象服务体系→建设和完善农村气象服务基础工程、农业可持续发展气象保障系统，提 升农民气象科技素质，增强农村气象防灾减灾能力。

　　(二)调整优化农业结构
　　1、积极发展优质农产品
　　继续扩大优质稻谷、优质专用小麦、优质专用玉米、优质小杂粮、优质马铃薯、高油高蛋白大豆种植面积，抓好优质棉花、“双低”油菜、“双高”糖料和无公害瓜果蔬菜生产，不断开发名特优新农产品。发展“一村一品”，加快培育专业村、专业乡、专业镇。
　　2、建设优势农产品产业带
　　重点建设长江流域和东北地区优质水稻产业带，黄淮海平原、长江下游、关中平原和河套平原优质专用小麦产业带，东北和黄淮海平原专用玉米产业带，东北大豆产业带，新疆、黄淮海和长江流域优质棉花基地，长江流域“双低”油菜产业带，广西、云南、广东、海南糖蔗基地，甘肃、内蒙古和宁夏马铃薯产业带，热带亚热带作物产业带。加强海峡两岸农业合作试验区和台湾农民创业园建设，扩大和深化两岸农业合作与交流，拓展两岸农业合作的深度和广度，逐步建立和完善两岸农业合作机制。
　　3、大力发展畜牧业
　　坚持把发展畜牧业作为农业结构战略调整的重点，优化布局结构，转变增长方式，加大扶持力度，加快发展步伐，做大做强畜牧产业。
　　按照“稳定牧区、发展农区、合理开发南方草山草坡”的总格局，以草食、节粮型畜禽为主，稳定发展生猪和家禽生产，加快发展肉牛肉羊生产，大力发展奶业，扶持南方地区发展水奶牛，鼓励发展特种养殖。东部沿海地区和无规定动物疫病区要加强畜产品出口基地建设，发展外向型畜牧业，提高我国畜产品的国际市场竞争力。中部地区要充分利用粮食和劳动力资源丰富的优势，加快现代畜牧业建设，提高综合生产能力。西部地区要稳定发展草原畜牧业，大力发展特色畜牧业。
　　推进畜牧业科技进步，积极利用生物技术，培育畜禽新品种。加快畜牧业科技成果转化，抓好畜禽品种改良、动物疫病诊断及综合防治、饲料配制和集约化饲养等技术的推广，提高畜牧业科技水平。积极发展饲料作物，大力发展饲料工业，重点扶持一批有发展潜力的大型饲料企业，加强饲料安全管理，开发高效、低毒、低残留的新型兽药，从源头上把好养殖产品质量安全关。转变畜禽饲养方式，推行健康养殖模式，加快建立一批标准化、规模化生产示范基地，扶持发展规模化养殖场和养殖小区，提高畜牧业养殖水平。
　　完善扶持畜牧业发展的政策措施，加强畜牧业规模化养殖小区水、电、路等公共基础设施建设，继续实施退牧还草工程，加大对西南岩溶地区种草养畜的扶持力度，保护和建设草原。扩大对畜牧业的财税支持，重点支持畜禽良种推广、种质资源保护、优质饲草基地和标准化养殖小区示范等方面的建设，继续清理畜禽养殖和屠宰加工环节不合理税费，继续实行对饲料产品的优惠税收政策，调整完善畜产品出口退税政策，对引进优良种畜禽、牧草种子继续免征进口关税和进口环节增值税。加大对畜牧业的金融支持，运用贴息等方式，引导和鼓励各类金融机构增加对畜牧业的贷款，鼓励社会资本参与现代畜牧业建设。
　　4、积极发展水产业
　　按照“保护资源、科学养殖、持续发展”的原则，继续执行海洋伏季休渔、长江春季禁渔和捕捞“双控”制度，严格控制近海捕捞强度，积极开展增殖放流，加大水生生物自然保护区建设，保护和恢复渔业资源。积极发展过洋性渔业，加快开拓大洋性渔业。积极推广生态养殖模式，加快发展名特优新水产品。鼓励发展休闲渔业。加强渔政渔港基础设施建设，积极构建“平安渔业”。
　　5、壮大林业产业体系
　　按照“保护优先、持续利用”的原则，积极发展速生丰产用材林、经济林和工业原料林，搞好木材精深加工，推广林纸、林板一体化，提高林产品的附加值。培育发展竹产业、花卉和林木种苗业、森林旅游业和野生动物驯养繁殖等林业产业。

专栏4　调整优化农业结构重点工程 　　以新疆为主的优质棉基地建设→继续建设新疆优质棉基地，完善良种繁育体系，扩 大商标准棉田规模，续建完善技术综合服务体系。在长江流域、黄河流域优势产区，建 设棉花生产基地。 　　长江流域优质油菜带建设→建设良种繁育及统一供种体系、小型农田水利工程，完 善病虫害防治和土肥监测等设施，逐步实现“双低”油菜规模化、标准化、优质化生 产。 　　糖料基地建设→重点支持广西、云南、广东、海南等甘蔗优势产区建设甘蔗生产基 地，改善田间灌溉条件，加快甘蔗新品种繁育和推广。 　　畜禽养殖小区示范工程→在优势区域建设一批畜禽养殖示范小区，规范养殖小区建 设和生产标准，重点抓好养殖小区畜禽良种、动物防疫和环保设施等建设，带动现代畜 牧业发展。 　　渔港工程→重点建设一批沿海中心渔港、一级渔港，提高渔业防灾减灾能力。 　　速生丰产用材林基地建设→积极推进速生丰产用材林基地建设。重点发展南方原料 林产业带、长江中下游原料林产业带、黄淮海平原工业原料林产业带。以国有林场和森 工企业为依托，大力建设各类珍稀树种培育基地。

　　(三)增加农民收入
　　1、挖掘农业内部增收潜力
　　适应人民生活水平提高和产业结构优化升级的需要，树立和运用大市场、大资源、大农业的理念，挖掘农业内部增收潜力，积极发展品质优良、特色明显、附加值高的优质农产品、特色农产品和专用农产品，扩大蔬菜、园艺等劳动密集型农产品出口，促进农业增产增效。优化农业内部结构，大力发展养殖业，带动粮食转化增值。适应农业食物保障功能强化、原料供给功能强化、就业增收功能强化和生态保护功能彰显、观光休闲功能彰显、文化传承功能彰显的新变化，积极开发农业多种功能。拓展农业的生态功能、观光功能和文化传承功能，积极发展“农家乐”等休闲农业、观光农业和旅游农业。开发农业新功能，在保障国家粮食安全的基础上，积极发展能源作物，拓宽农业增收渠道。
　　2、大力推进产业化经营
　　认真落实扶持龙头企业发展的政策措施，发挥农民专业合作组织的纽带作用，完善龙头企业与农户的利益共享、风险共担机制，引导和鼓励龙头企业通过建立标准化生产基地、发展订单农业等形式与农民建立稳定的产销关系，培育发展“龙头企业＋农户”、“龙头企业＋农民专业合作经济组织＋农户”和“农产品行业协会＋龙头企业＋农民专业合作经济组织＋农户”等新型农业经营模式，逐步将一家一户的分散经营纳入规模化、市场化的现代生产经营体系。
　　3、加快发展农村二、三产业
　　以资源为基础，以市场为导向，以体制机制创新为动力，加快发展农村二、三产业，促进农村富余劳动力就近就地转移就业。
　　加快发展农村工业。大力发展农副产品加工业，延长农业产业链条，构建生产、加工、销售有机结合的现代农业产业体系，提升农产品深加工水平和综合利用水平，提高农业资源的利用效率和农产品的附加值。重点发展粮油加工业、糖料加工业、果蔬加工业、肉类水产加工业、乳制品加工业和农业特色资源加工业等农副产品加工业，扶持发展一批重点骨干农副产品加工企业。结合新农村建设，加快发展农村建筑业。引导农村工业向县城和小城镇集中，促进产业集聚。落实促进乡镇企业发展的政策措施，加大对规模以上乡镇企业技术改造的支持力度，提高乡镇企业技术装备水平，促进乡镇企业产品更新换代和产业优化升级。
　　大力发展农村第三产业。重点完善农村流通体系，积极推进农产品批发市场升级改造，加快发展农产品保鲜、储藏、配送、运输等冷链物流设施，完善鲜活农产品“绿色通道”网络。大力发展农村连锁经营、电子商务等现代流通方式，加快建设“万村千乡市场”、“双百市场”、“新农村现代流通网络”和“农村商务信息服务”等工程。加快供销社经营网络升级改造，鼓励城市商业网点向农村延伸。积极发展农村集贸市场，规范发展期货市场，稳步发展农资市场。加强农资市场监管，依法打击制售假冒伪劣商品。加快发展农村交通运输业、餐饮业和旅馆服务业。积极发展乡村旅游。
　　4、发展壮大县域经济
　　加快发展小城镇。重点抓好县城和中心建制镇的规划和建设，健全小城镇投融资机制，从财政、金融、税收和公共品投入等方面为小城镇发展创造条件，加快改变小城镇基础设施落后的局面。大力发展小城镇经济，坚持把发展小城镇同调整乡镇企业布局、发展农村二、三产业、完善农村市场体系等结合起来，以小城镇为依托，以产业为基础，以企业为载体，加快农村二、三产业向小城镇集中，带动农业富余劳动力向非农产业和小城镇转移，引导农村人口到小城镇定居，加快形成企业集群、产业聚集、人口集中、市场扩大的良性互动机制，促进小城镇快速健康发展，带动县域企业集群和产业集聚，培育县域主导产业，发展壮大县域经济。
　　大力发展民营经济。认真落实国务院《关于鼓励支持和引导非公有制经济发展的若干意见》，在投融资、资源使用、人才政策等方面，对农村个体工商户和私营企业给予支持，扶持发展农村个体私营等非公有制经济，促进民营经济快速发展，增强县域经济发展活力。
　　5、大力发展劳务经济
　　认真落实《[国务院关于解决农民工问题的若干意见](https://www.pkulaw.com/chl/de43d86a655f08cfbdfb.html)》，进一步改善农民外出务工环境，维护农民工合法权益，促进农民进城务工。改革城乡分割的就业管理体制，取消针对农民跨区域就业、进城务工的歧视性规定和不合理限制，加快建立城乡统一的劳动力市场和就业制度，为城乡劳动者提供平等的就业机会和服务。深化户籍管理制度改革，逐步地、有条件地解决长期在城市就业和居住农民工的户籍问题。合理调整和严格执行最低工资制度，落实小时最低工资标准，建立农民工工资合理增长机制和工资支付保障制度，依法查处拖欠克扣农民工工资和随意延长工时的行为。完善农民工劳动合同制度，加强职业安全卫生保护，改善劳动环境。逐步建立农民工社会保障制度，依法将农民工全部纳入工伤保险范围，加快解决农民工大病医疗保障问题，探索符合农民工特点的养老保险办法。帮助农民工解决住房、子女上学等实际困难，保障农民工合法权益。制定扶持政策，鼓励和支持外出务工农民带技术、带资金回乡创业，带动农民致富。
　　加强对农民工的培训和教育。在搞好基本权益保护、法律知识、城市生活常识等引导性培训的基础上，重点抓好对农民工的职业技能培训和鉴定，以适应新的就业岗位的需要。继续实施“农村劳动力转移培训阳光工程”，五年培训农民工5000万人以上，重点加强职业技能培训。
　　6、继续做好扶贫工作
　　坚持开发式扶贫方针，加大扶贫工作力度，创新扶贫工作方式。逐步增加财政扶贫资金、以工代赈等专项扶贫投入，加强和完善项目管理，提高扶贫资金使用效益。从实际出发，因地制宜地开展扶贫开发工作。对具备基本生存条件的贫困地区，继续以改善基本生产生活条件为中心，重点解决好贫困人口的吃饭、饮水和增加收入问题。对缺乏基本生存条件的贫困人口，有计划地实行易地扶贫搬迁工程。扎实推进整村扶贫计划，集中财力改善贫困地区的生产生活条件，加强贫困地区农村劳动力转移培训工作，扶持贫困地区发展特色产业及龙头企业，扩大贫困农民脱贫致富途径。高度重视农村特困户的扶贫和救济工作，对不具备基本劳动能力的贫困人口，通过建立最低生活保障，实行救济扶贫。对特困户子女通过寄宿教育、家庭寄养、社会托养、减免非义务教育收费等方式，改善其教育条件和成长环境。继续动员社会力量参与农村扶贫开发事业，弘扬中华民族扶贫济团的传统美德。

专栏5　增加农民收入重点工程 　　农业产业化经营项目→采取投资参股、贷款贴息等方式，建设高效种植和养殖基 地，加大对农产品加工龙头企业、农产品储藏保鲜和产地批发市场的扶持能力，延长农 业产业链，促进农业增产增效。 　　农产品深加工示范工程→重点支持一批技术含量高、创新能力强、市场前景好、辐 射带动面广，具有一定国际竞争力的农产品深加工骨干企业，推动农产品加工业向深、 精、特、优方向发展。 　　农村市场流通基础设施工程→重点对500家骨干农产品批发市场交易场地、交易棚 厅、水电道路、卫生保洁进行升级改造，完善批发市场储藏、保险、加工分选包装、市 场管理、市场信息采集发布、质量安全监测等功能，建设鲜活农产品冷链物流设施；支 持农村生活日用品批发市场、农资连锁经营、配送中心等建设。 　　经济综合开发示范镇工程→继续在全国选择部分重点镇，通过扶持产业开发，完善 基础设施，健全市场体系和服务体系，引导企业集群和产业集聚，扶持发展小城镇经 济。 　　农村劳动力转移就业→加强农村劳动力技能培训、鉴定、就业服务和维权服务能力 建设，为外出务工农民免费提供法律咨询、就业信息、就业指导和职业介绍。 　　以工代赈工程→加强贫困地区基本农田、小型农田水利、乡村道路、草场建设、饮 水安全和小流域等基础设施建设，改善贫困地区生产生活条件，促进农民增收。 　　易地扶贫搬迁试点工程→有计划地对不适宜人类生存地区的贫困人口实施搬迁，缓 解迁出地人口压力，改善迁入地生产生活条件，拓展脱贫致富渠道，恢复和保护迁出地 生态环境。

　　(四)加强生态环境建设
　　1、加强生态保护
　　坚持保护优先、开发有序，以控制不合理的资源开发活动为重点，强化对水源、土地、森林、湿地、草原、海洋等自然资源的生态保护，逐步实现生态建设重点从事后治理向事前保护转交，从人工建设为主向人工建设与自然恢复并重转变，从源头上扭转生态环境恶化的趋势。以主体功能区划分为依据，加强对自然生态的监测评估和保护，对划为限制开发区域的大小兴安岭森林生态功能区、长白山森林生态功能区、川滇森林生态及生物多样性功能区、秦巴生物多样性功能区、藏东南高原边缘森林生态功能区、新疆阿尔泰山地森林生态功能区、青海三江源草原草甸湿地生态功能区、新疆塔里木河荒漠生态功能区、新疆阿尔金草原荒漠生态功能区、藏西北羌塘高原荒漠生态功能区、东北三江平原湿地生态功能区、苏北沿海湿地生态功能区、四川若尔盖高原湿地生态功能区和甘南黄河重要水源补给生态功能区等限制开发区域，加强生态修复和环境保护，引导超载人口逐步有序转移，控制人为因素对自然生态的干扰和破坏。对划为禁止开发区域的国家级自然保护区、世界文化自然遗产、国家重点风景名胜区、国家森林公园、国家地质公园等，实行强制性保护，控制人为因素对自然生态的干扰，严禁不符合主体功能定位的开发活动。依法全面落实开发建设项目水土保持“三同时”制度。加大对外来入侵生物的防治力度，防止外来入侵生物对生态系统的破坏。加强森林草原防火工作。按照谁开发谁保护、谁受益谁补偿的原则，加快建立生态补偿机制。
　　2、强化综合治理
　　根据《全国生态环境建设规划》的总体部署，以目前生态环境脆弱、对全局生态环境影响大的地区为重点，以小流域为单元，按照因地制宜、综合治理的思路，宜林则林，宜草则草，坚持工程措施、生物措施和农艺措施相结合，水土保持、造林种草等相配套，切实抓好京津风沙源区、青海三江源自然保护区、青海湖地区、三峡库区、“三北”风沙区、北方草原区、黄土高原区、南方岩溶地区、东北黑土漫川漫岗区等重点区域和黄河中上游、长江中上游、塔里木河、黑河、石羊河等重点流域的生态环境保护与建设。抓紧编制岩溶地区石漠化综合治理规划大纲，尽快启动石漠化综合治理工程，加快治理石漠化地区。加强南水北调工程水源地及沿线生态环境保护与建设。
　　3、抓好重点工程
　　坚持以生态建设为主的林业发展战略，按照“西治、东扩、北休、南用”的思路，优化林业生态建设布局，继续抓好天然林资源保护、退耕还林、防护林体系、湿地保护与修复、野生动植物保护及自然保护区建设等重点林业生态建设工程。在已经大规模开展退耕还林还草的地区，通过建设基本口粮田、发展农村能源、培育后续产业、实行生态移民、抓好禁牧舍饲等配套措施，巩固退耕还林还草成果。
　　加快实施西部天然草原退牧还草工程．抓好草场围栏，加快草场改良，建设人工饲草基地，发展牧区水利，加强对草原生态的保护与建设，防止草原退化和沙化。引导牧民定居。
　　抓好水土保持工程建设。继续对长江上中游、黄河上中游、京津风沙源区、首都水源区、晋陕蒙砒砂岩区、东北黑土区、珠江上游南北盘江石灰岩区等水土流失严重地区实施重点治理。加强对坡耕地的综合治理，启动坡耕地水土流失综合整治工程。积极推进水土保持生态修复工作和水源区水土流失防治及面源污染防治工作。鼓励支持农户、专业队及其它社会法人通过承包、租赁、股份合作等方式参与水土流失治理，推动农村“四荒”水土流失治理工作。

专栏6　生态保护和建设重点工程 　　天然林资源保护工程→对工程区内9418万公顷天然林和其他森林实行全面有效管护，在 长江上游、黄河上中游工程区造林579万公顷。 　　退耕还林还草工程→在长江、黄河流域水土流失以及北方风沙地区等继续实施退耕还林 还草。 　　退牧还草工程→在内蒙古东部、内蒙古甘肃宁夏西部、青藏高原东部、新疆北部四大片 区，通过建设草原围栏、补播重度退化草地，实行禁牧、休牧、轮牧等措施治理严重退化草 原。 　　水土保持工程→新增水土流失治理面积2500万公顷；实施石羊河流域综合治理。 　　京津风沙源治理工程→对严重沙化耕地和陡坡耕地退耕还林还草34万公顷，配套宜林荒 山荒地造林29万公顷。同时采取人工造林、飞播造林、封育、草地治理、小流域综合治理、 水源及节水灌溉、生态移民等措施，对工程区内的沙化土地进行综合治理。 　　青海三江源自然保护区生态保护和建设工程→退牧还草644万公顷，退耕还林还草0．65 万公顷，封山育林、沙漠化土地防治、湿地保护、黑土滩治理80万公顷，鼠害治理209万公 顷，水土流失治理5万公顷。 　　石漠化地区综合治理→通过植被保护，退耕还林，封山、造林、种草及草地治理，大力 发展草食畜牧业，合理开发利用水土资源，搞好蓄水保土工程，加强基本农田建设，加快农 村能源建设，易地扶贫搬迁等措施，加大石漠化地区治理力度。 　　防护林体系工程→建设“三北”防护林体系四期工程，长江、珠江防护林和太行山绿 化、平原绿化及沿海防护林体系工程。推进三峡库区绿化带建设。 　　野生动植物保护及自然保护区建设→建设和完善一批自然保护区，继续实施对极度濒危 野生动植物物种的拯救工程。 　　湿地保护与修复→建设222个湿地保护区，其中国家级湿地保护区49个，通过对水资源 的合理调配和管理等措施恢复重要湿地。 　　森林防火和病虫害防治体系建设→加强林区道路阻隔、预警通信、了望监测等森林防火 基础设施建设，提高森林火灾综合防控能力。加强森林病虫害防治基础设施建设，加强对松 材线虫病、美国白蛾、杨树天牛等危险性或大面积发生的林业有害生物防治。建立林业有害 生物预防预警和应急体系。 　　草原防火工程→重点建设内蒙古、新疆、黑龙江、甘肃、青海、河北等13省(区)及新疆 生产建设兵团草原防火物资保障系统及火灾监测预警指挥系统。

　　(五)改善农村生产生活条件
　　1、加强农业和农村基础设施建设
　　认真落实中央提出的把国家对基础设施建设投入的重点转向农村的重大战略部署，突出工作重点，切实抓好农民最关心、最急需、最现实的饮水安全、农田水利、农村道路、农村沼气、农村电力、农村垃圾和污水处理等基础设施建设。
　　加快农村饮水安全工程建设。在巩固人畜饮水解围成果基础上，进一步加大农村饮水安全工作力度，组织实施好农村饮水安全工程，解决1．6亿农村人口的饮水安全问题。重点解决饮用水高氟、高砷、苦咸、污染及微生物病害等严重影响身体健康的水质问题，以及局部地区的严重缺水问题，优先解决人口较少民族、水库移民、血吸虫病区和农村学校的饮水安全问题。东部发达地区要充分发挥地方政府的积极性，率先解决农村饮水安全问题，有条件的地方实现城乡统筹供水。国家重点支持中西部地区农村饮水安全工程建设。提倡饮用水和其它生活用水分质供水。在重要饮用水水源地，采取水土保持生态缓冲带、生物净化等技术，加大对农药、化肥等面源污染的控制力度，确保人民群众饮水安全。
　　加强农田水利基础设施建设。继续开展大型灌区续建配套和节水改造，完善灌排设施，加强干支渠和末级渠系建设，提高灌溉水的利用效率和灌区生产能力。在水资源相对丰富和土地条件相对较好的地区，结合水源工程建设，适量开发建设后备灌区。实施排涝泵站更新改造工程，提高中部低洼地区的排涝能力。创新机制，加大投入，引导农民投工投劳，切实加强田间灌排工程、小型灌区、非灌区抗旱水源工程等小型农田水利建设。五年新增工程节水灌溉面积1000万公顷，净增有效灌溉面积133-200万公顷。
　　加快农村能源建设步伐。大力普及农村沼气，重点建设以沼气池和改圈、改厕、改厨为基本内容的农村户用沼气工程，力争新增农村沼气用户2200万户，鼓励和支持规模化畜禽养殖场(含养殖小区)建设大中型沼气工程，有效解决农民生活用能、农村人畜粪便污染及农村环境脏乱差等问题。重视并加强沼气技术支撑及服务体系建设。在保护生态环境的基础上，有序开发小水电资源，按规划继续实施水电农村电气化县和小水电代燃料生态保护工程。因地制宜，积极发展适合各地特点的太阳能、风能和生物质能等清洁能源，建设一批绿色能源示范县。配合发展燃料乙醇和生物柴油，建设能源作物供应基地。
　　加大农村公路建设力度。按照《农村公路建设规划》的总体部署，加快农村公路改造和建设，到2010年基本实现全国所有乡镇通沥青(水泥)路，东、中部地区所有具备条件的建制村通沥青(水泥)路，西部地区基本实现具备条件的建制村通公路(西藏自治区视建设条件确定)。建立健全农村公路管理养护体制和运行机制。
　　继续抓好农村电网改造。加快完善中西部农村电网，提高农网改造覆盖率，扩大电网供电人口覆盖面。在电网延伸供电不经济的地区，根据地区特点，积极发展小水电、太阳能光伏发电和风力发电，解决农村无电人口的基本用电问题。
　　大力发展气象事业。加快实施气象卫星和新一代天气雷达组网建设，健全气象服务业务系统，提高气象灾害监测预警能力、气候变化应对能力、气候资源开发利用能力和人工防雹增雨(雪)能力。

专栏7　农业和农村基础设施建设重点工程 　　大型灌区续建配套节水改造工程→开展灌区续建配套与节水改造，提高灌溉水利用效率 和灌区生产能力。 　　中部四省大型排涝泵站改造工程→更新改造湖南、湖北、江西、安徽四省已有大型排涝 泵站。 　　中型灌区节水配套改造→加强干支渠配套完善和更新改造，创新工程管护运行机制，为 改造中低产田提供中型灌排工程。 　　农村饮水安全工程→解决1．6亿农村居民饮用高氟水、高砷水、苦咸水、污染水和血吸 虫病区、微生物超标等水质不达标及局部地区严重缺水问题。 　　农村公路建设→新建和改建农村公路约120万公里，东、中部地区重点建设建制村通沥 青(水泥)路，西部地区重点建设乡镇通沥青(水泥)路和建制村通公路。 　　农村沼气工程→通过“一池三改”等措施，新增农村沼气用户2200万户，鼓励和支持规 模化畜禽养殖场和养殖小区建设大中型沼气工程。 　　送电到村和绿色能源县工程→建成50个绿色能源示范县，利用电网延伸、风力发电、小 水电、太阳能光伏发电等，解决无电人口基本用电问题。 　　气象卫星及其应用工程→建设气象卫星的研制、发射和测控工程以及地面应用系统。 　　新一代天气雷达组网工程→在全国完成新一代天气雷达系统的布点建设，建立国家级和 区域级雷达信息高速实时传输系统，以及国家和省两级雷达资料海量存储检索系统等。

　　2、搞好村镇规划和建设
　　适应农村人口转移和村庄减少的趋势，结合农村基础设施建设和社会事业发展，按照布局合理、节约土地、因地制宜、突出特色的要求，科学编制县域村镇体系规划和村镇建设总体规划，优化村镇布局。加大对有特色的农村建筑风貌的保护力度。重点建设中心镇、中心村，积极稳妥地引导分散居住的农民向中心镇、中心村集中。山区和丘陵地区，也可以自然村和村民小组为单位，引导散居、独居农户适当集中。结合生态移民和重大工程项目建设，有计划地引导农民搞好移民搬迁和村庄迁建。在引导农民适当集中居住的过程中，一是要充分尊重农民意愿，二是要依法保障农民的权益。
　　扎实推进村庄整治工作。充分利用现有房屋、设施和自然条件，重点完善农民生产生活最急需的公共设施。进一步研究解决好一些村的村内道路建设主体不清、建设资金缺乏等问题。加强宅基地规划和管理，引导农民按规划建房，保护好农民对于宅基地的合法权益。结合农村小康环保行动计划，切实加强农村环境综合治理，动员组织农民清理农村垃圾污染，帮助解决一些农民的住宅与畜禽圈舍混杂问题。加强村庄绿化工作，改善农村人居环境。经济基础较好、人口比较集中的地区，在尊重农民意愿的前提下，推进农村社区建设。
　　(六)大力发展农村社会事业
　　1、优先发展农村教育
　　普及和巩固农村义务教育。逐步将农村义务教育全面纳入公共财政保障范围，建立中央和地方分项目、按比例分担的农村义务教育经费保障机制。2007年起免除全国农村义务教育阶段中小学学生学杂费，并对贫困家庭学生免费提供课本和补助寄宿生生活费。完成西部“两基”攻坚计划和农村中小学现代远程教育工程，实施农村初中校舍改造工程，切实改善农村中小学办学条件。实施农村教师培训工程，力争在五年内使中西部地区50％的农村教师得到一次专业培训。实施“农村义务教育阶段学校教师特设岗位计划”，鼓励和引导城市教师及具有教师资格的其他人员到农村中小学任教。大力发展农村职业技术教育，加快培养新型农民和农村实用人才，加强对农民的职业技能培训和鉴定，逐步把农民培训纳入全社会教育体系，重点建设1000所县级职教中心和1000所中等职业学校，加大绿色证书培训力度和农村实用人才培训工程实施力度，培养有文化、懂技术、会经营的新型农民，提高农民科技文化素质。
　　2、加强农村公共卫生和基本医疗
　　强化政府责任，加强政府在医疗卫生领域的公共服务职能，建设覆盖城乡居民的基本卫生保健制度，为农民群众提供安全、有效、方便、价廉的公共卫生和基本医疗服务。健全县、乡、村三级农村医疗卫生服务网络，到2010年基本完成以乡镇卫生院为重点的农村卫生服务体系基础设施建设任务。加强农村传染病、地方病和人畜共患疾病的防治工作，提高农村应对突发公共卫生事件的能力。加强农村卫生人才队伍建设，鼓励城市医务人员到农村服务。整顿农村药品和医疗器械流通秩序，保障农民用药安全。
　　3、加快农村公共文化建设
　　加快广播电视村村通工程建设步伐，到2010年基本实现20户以上已通电自然村通广播电视。加强全国文化信息资源共享工程建设，力争到2010年实现县县有分中心、乡镇有基层分中心、村村有基层服务点。继续实施农村电影放映工程，到2010年基本实现全国农村每村每月放映一场电影的目标。加强乡镇综合文化站建设。落实向广大村民配送图书、报刊、音像出版物活动，到2010年全国建设20万个“农家书屋”。做好农村非物质文化遗产保护工作，继承和发展优秀民族民间传统文化。实施农民体育健身工程，推进乡村体育设施建设，到2010年为l0万个行政村建成简易硬化篮球场等体育设施，为农村开展全民健身活动创造条件。
　　4、稳定农村低生育水平
　　坚持计划生育基本国策，完善现行生育政策。消除低生育水平反弹的各种隐患，落实目标责任制度，严格控制农村人口数量过快增长。健全利益导向机制，完善计划生育技术服务基本项目免费制度，在全国范围实施农村计划生育家庭奖励扶助制度，加大“少生快富”扶贫工程实施力度。实施农村计划生育服务设施建设工程，以中西部地区县、乡计划生育技术服务站建设为重点，形成以县级服务站为龙头、中心乡镇服务站为骨干、流动服务车为纽带的农村基层计划生育服务体系。倡导优生优育，加大出生缺陷干预力度。严厉打击非医学需要的胎儿性别鉴定和选择性别终止妊娠的行为，深入开展关爱女孩行动和婚育新风进万家活动，综合治理出生人口性别比失调问题。
　　5、加快建立农村社会保障制度
　　抓紧在农村建立最低生活保障制度，从各地实际出发，科学确定与各地经济社会发展水平、财力状况和当地生活水平相适应的保障标准和范围，坚持公开、公平、公正的原则，实行民主评议、社会公示、动态管理等形式，将符合条件的农村贫困人口纳入保障范围，重点保障病残、年老体弱、丧失劳动能力等生活常年困难的农村居民。做好农村最低生活保障制度与其他农村社会保障政策和补助措施的衔接工作。迸一步完善农村“五保户”供养、特困户生活救助、农村灾民救助等农村社会救助体系。加快推进新型农村合作医疗，到2008年在全国基本建立新型农村合作医疗制度，逐步提高保障水平。完善农村医疗救助制度。有条件的地区加快探索建立多种形式的农村社会养老保险制度。
　　6、加强农村民主政治建设和精神文明建设
　　结合“八荣八耻”教育，提倡讲文明、树新风，积极开展创建“文明村镇”、“文明户”活动，经常性开展科技、文化、卫生“三下乡”和群众文化体育活动，引导农民崇尚科学、抵制迷信、移风易俗，努力形成科学健康的生活方式和文明向上的社会风貌。

专栏8　农村社会事业建设重点工程 　　“两基”攻坚农村寄宿制学校建设→重点支持尚未实现“两基”的中西部农村地区，新 建和改扩建7700多所农村寄宿制学校。 　　农村中小学现代远程教育→为中西部地区3.75万所农村初中建设计算机教室、为38.4万 所农村小学配备卫星教学接收设备、为11万个小学教学点配备教学光盘播放设备和成套教学 光盘。 　　中西部农村初中改造→推动未纳入“两基”攻坚计划实施范围的中西部地区农村初中校 舍改造，改善办学条件，提高学生巩固率和寄宿率。 　　农村卫生服务体系→以中西部地区乡镇卫生院为重点，同步建设县医院、县妇幼保健机 构、县中医院(民族医院)、村卫生室。 　　农村计划生育服务体系→建设县级服务站、乡镇中心服务站，购置计划生育设备和流动 服务车等。 　　村村通广播电视→基本实现20户以上已通电自然村通广播电视。 　　农村电影放映→基本实现全国农村一村一月放映一场电影。 　　乡镇综合文化站建设→基本实现全国乡镇均建有综合文化站。 　　文化信息资源共享→推进文化资源数字化，以农村为重点促进文化信息资源共享。 　　农民体育健身工程→在10万个行政村建成简易硬化篮球场等体育设施。 　　农村邮电通信服务体系→基本实现村村通电话，乡乡能上网。

四、保障措施

(一)切实保护好水土资源
　　坚持最严格的耕地保护制度，切实保护好耕地资源特别是基本农田。完善土地利用总体规划，加强土地用途管制，对农用地转用计划实行指令性管理，建立健全建设用地定额标准，从严从紧控制建设用地过快增长，促进土地节约和集约使用。清理和规范各类开发区，严禁占而不用或占多用少。加强农村集体建设用地管理，禁止擅自通过“村改居”等方式将农民集体所有土地转为国有土地，严禁“以租代征”擅自将农用地转为建设用地。改革和完善农村宅基地审批制度，禁止城镇居民在农村购置宅基地，推广使用新型墙体材料，引导农民建设节能省地型住房，引导各类企业向镇区集中，减少农村企业和宅基地占地规模。严格落实地方各级政府对规划确定的本行政区域内的基本农田面积和耕地保有量负责制。切实保护好基本农田，加快农村集体土地所有权的确权登记发证工作，将基本农田落实到地块和农户。任何单位和个人不得擅自占用基本农田或者改变基本农田用途，禁止占用基本农田挖塘养鱼、毁田烧砖、植树造林或进行其它破坏耕作层的活动，禁止以建设“现代农业园区”或者“设施农业”等名义，占用基本农田变相从事房地产开发。建立基本农田年度检查和动态监管制度，确保基本农田总量不减少、质量不降低、用途不改变。严格执行耕地占补平衡制度，加大土地整理复垦力度。对补充耕地的数量、质量实行按等级折算，防止占多补少、占优补劣。统筹考虑生态建设与粮食生产的关系，合理安排生态退耕规模。
　　加强水资源管理。健全流域管理与区域管理相结合的水资源管理体制，逐步建立以水权管理为基础的水资源管理制度。加强水资源的公平分配、优化配置、高效利用和节约保护，统筹安排好生活、生产和生态用水，统一调配上下游和地表、地下水，初步实现国家对重要江河水资源和主要平原区地下水的有效监测。科学开展人工增雨(雪)作业，提高空中水资源的开发利用水平。继续进行海水淡化技术的开发和利用。加强地下水资源管理，严格限制超采滥采地下水。合理公平分配水权，实行用水总量控制和定额管理相结合，制定和完善主要江河的水量分配方案以及旱情紧急情况下的水量调度预案，初步建立国家水权制度。加强水污染防治工作，严格保护地表和地下水，逐步扭转水环境恶化趋势，维护河流健康生命。
　　(二)加强对农业和农村的支持与保护
　　按照中央关于把国家对基础设施建设投入的重点转向农村的部署和要求，下决心调整国民收入分配结构，加大财政支农力度，扩大公共财政覆盖农村的范围，提高政府对农村的公共服务水平。国家财政支出和预算内固定资产投资，要按照存量适度调整、增量重点倾斜的原则，不断增加对农业和农村的投入。各级政府要把基础设施建设和社会事业发展的重点转向农村，财政新增教育、卫生、文化等社会事业经费和固定资产投资增量主要用于农村，逐步加大政府土地出让金用于农村的比重。抓紧建立“三农”投入稳定增长机制，积极调整财政支出结构、固定资产投资结构和信贷投放结构，中央和县级以上地方财政每年对农业总投入的增长幅度要高于其财政经常性收入的增长幅度，研究制定土地出让金用于农业土地开发的具体办法和措施，尽快形成新农村建设稳定的资金来源。引导地方政府加大支农投资力度，逐步改变一些地区农业和农村基础设施建设过多依赖中央政府投资的状况。
　　调整优化政府支农投资结构，在继续加强水利等基础设施建设和生态环境建设的同时，重点向提高农业综合生产能力和农产品国际竞争力的方面倾斜，向能够改善农村生产生活条件和直接促进农民增收的项目倾斜，向中西部地区和贫困地区倾斜。创新政府支农投资方式，采取投资补助、资本金注入、财政贴息等多种投资方式，发挥政府支农投资的引导作用，吸引社会资金投向农业。整合政府支农投资，完善中央支农投资协调机制，建立以规划定项目、以项目定投资的政府支农投资管理制度，集中力量办大事，提高政府支农投资效率。
　　完善和加强国家对农业和农民的支持保护体系。加大对种粮农民的补贴力度，在稳定和完善粮食直补、开展对种粮农民柴油、化肥等农业生产资料增支综合直补的基础上，抓紧建立粮食综合补贴制度。增加良种补贴和农机具购置补贴规模。研究制定超载减畜补贴、农村小型公益基础设施建设补贴等政策。逐步建立促进粮食生产稳定发展和保护种粮农民利益的长效机制。适应农业生产发展和市场变化的需要，不断加大补贴力度，完善补贴方式，建立和完善国家对农业和农民的补贴政策体系。继续实行中央财政对粮食生产大县的奖励政策，建立和完善对粮食主产区的补偿机制。
　　引导社会资金支持“三农”建设。加快推进农村金融改革和发展，采取有效措施。加强对农村的金融服务，为建设社会主义新农村提供有力的金融支持。加快建立健全适应“三农”特点的多层次、广覆盖、可持续的农村金融体系。健全农村金融组织体系，充分发挥商业性金融、政策性金融、合作性金融和其他金融组织的作用。推进农村金融组织创新，适度调整和放宽农村地区金融机构准入政策，降低准入门槛，鼓励和支持发展适合农村需求特点的多种所有制金融组织，积极培育多种形式的小额信贷组织。引导邮政储蓄等资金返还农村。在贫困地区先行开展发育农村多种所有制金融组织的试点。积极安排国外优惠贷款支持“三农”建设。大力推进农村金融产品和服务创新，积极发展农业保险，按照政府引导、政策支持、市场运作、农民自愿的原则，建立完善农业保险体系。加快建立各行各业支持农业生产、全社会支持新农村建设的激励机制，鼓励社会力量积极参与农村基础设施建设，引导广大农民积极投身新农村建设，构建政府投入为导向、农民和企业投入为主体、其它资金投入为补充的农业投融资机制，多渠道增加农业和农村投资。
　　(三)深化农村改革开放
　　稳定并完善以家庭承包经营为基础、统分结合的双层经营体制。切实保护农民的土地承包经营权，依法引导和规范土地承包经营权流转，促进有条件的地方发展多种形式的适度规模经营。加快农村土地承包纠纷仲裁立法进程，依法建立健全土地承包纠纷仲裁体系，不断完善纠纷调处机制。
　　以促进农村上层建筑变革为核心，实行农村综合改革。积极稳妥地推进乡镇机构改革，按照建设服务型、法制型政府的要求，切实转变乡镇政府职能，把乡镇工作重点从直接抓生产经营、催种催收、招商引资等具体事务转到对农户和各类经济主体进行示范引导、提供公共服务和营造发展环境上来，加强社会管理和公共服务职能。整合乡镇事业站所，创新乡镇事业站所运行机制，精减机构和人员，妥善安置分流人员。有条件的地方，积极稳妥地做好乡镇、村组撤并工作，构建合理的乡村治理结构，建立精干高效的农村基层行政管理体制。加快农村义务教育管理体制改革，建立和完善政府投入办学、各级责任明确、财政分级负担、经费稳定增长的农村义务教育经费保障机制。调整农村学校布局，优化农村教师队伍，合理配置城乡教育资源，提高农村义务教育经费保障水平。改革县乡财政管理体制，有条件的地方要加快推进“省直管县”财政管理体制和“乡财县管乡用”财政管理方式的改革，加快建立规范的财政转移支付制度，完善中央缓解县乡财政困难的奖补办法，完善财力与事权相匹配的省以下财政管理体制。按照“制止新债、锁定旧债、明确责任、分类处理、逐步化解”的原则，妥善解决乡村债务问题，建立逐步化解乡村债务的有效机制。在新农村建设中，决不能盲目举债搞建设，形成新一轮乡村负债。创新农民负担监督管理机制，防止农民负担反弹，维护农民合法权益。
　　深化国有农场税费改革和管理体制改革，加快国有农场土地确权工作，积极培育现代化家庭农场和股份制农业经营公司。加快推进农村集体林权制度改革，明晰林地使用权和林木所有权，放活经营权，落实处置权，继续搞好国有林区林权制度改革试点，逐步建立“产权归属清晰、经营主体到位、责权划分明确、利益保障严格、流转顺畅规范、监管服务有效”的林业产权制度。
　　加快土地征用制度改革，严格区分公益性建设用地和经营性建设用地，从严从紧控制公益性用地，经营性用地出让要逐步实行招标、拍卖、挂牌出让等市场化方式。积极推进征地统一年产值标准或区片综合地价，征地补偿做到同地同价。建立征地和土地出让最低价格制度，被征地农民的社会保障费用要纳入征地补偿安置费用，合理提高征地补偿标准，积极推进土地资源的市场化配置，运用价格机制抑制多占、滥占和浪费土地。积极搞好水权制度改革，探索建立水权分配、登记、转让等各项管理制度。完善水资源有偿使用制度，改革水价形成机制，促进水资源的可持续利用。加快建立能够体现水土资源稀缺程度、保护农民利益的价格形成机制。
　　创新农村经济管理体制和经营机制，增强农村经济发展活力。创新农村基础设施建设机制和管理体制，吸引社会资金建设农村基础设施。积极推进农村小型水利工程产权制度改革，提倡采取拍卖、租赁、承包经营等方式创新经营机制，调动广大农民和社会法人参与小型水利工程建设和管理的积极性。推进乡镇企业股份制和股份合作制改造，鼓励有条件的乡镇企业建立现代企业制度。
　　扩大农业对外开放。加快实施农业“走出去”战略。加强农产品出口基地建设，集中力量建设一批出口潜力大、基础条件好、与主要出口市场相适应的农产品出口基地。搞好对农产品出口的信贷和保险服务，支持农产品出口企业在国外注册品牌，开拓市场，努力扩大优势农产品出口。支持企业、农民到国外从事农业开发和农产品贸易，拓展农业发展空间。加强农业国际合作与交流，积极引进国外技术、人才、资金和服务，促进农业生产发展和技术升级，提高农业国际竞争力。
　　(四)大力发展农民专业合作经济组织
　　发展现代农业，解决一家一户小生产与大市场的矛盾，完善农业社会化服务体系，创新农业经营体制，提高农民组织化程度和市场适应能力，以及保护农民合法权益等，都需要发展农民专业合作经济组织。认真贯彻落实《[农民专业合作社法](https://www.pkulaw.com/chl/b7e1ba41370a9d20bdfb.html)》，在市场准入、融资条件、政策支持等方面创造平等竞争的环境，支持农民按照自愿、民主的原则发展多种形式的专业合作经济组织，支持农民专业合作经济组织开展加工、运输、销售、生产资料供应、技术培训、农技推广等服务，对农民专业合作经济组织及其所办加工、流通实体实行鼓励和优惠政策。国家支持发展农业和农村经济的建设项目，可以委托和安排有条件的有关农民专业合作社实施。充分发挥农民专业合作经济组织的纽带作用及在政策执行和市场经营活动中的中介作用，为龙头企业和广大农户提供服务。
　　加强对农产品行业协会的扶持和引导。依托重点产品、重点领域，加快培育区域性和全国性农产品行业协会。结合政府职能转变，逐步将可由行业协会协调或管理的事权和职能逐步转移给行业协会，充分发挥行业协会在制定标准、产品推介、应对纠纷、维护权益、咨询服务及行业自律等方面的作用。
　　(五)加强和改善对农村经济的宏观调控
　　坚持把巩固和加强农业基础地位、确保国家粮食安全、促进农民增收、建设社会主义新农村作为宏观调控的重要任务。发展改革、财政金融及涉农部门要加强对农业和农村经济运行情况的监测和预警，及时提出调控的方向和措施，充分发挥经济杠杆的作用，综合运用各种调控手段，确保主要农产品供求基本平衡、农村经济稳步发展和农民收入持续增长，避免出现大起大落。
　　切实加强对粮食生产和粮食市场的宏观调控。高度重视粮食生产，加强粮食生产能力建设，建立粮食主产区和主销区利益协调机制，以市场化为基本取向，完善粮食最低收购价政策，充分发挥价格对粮食生产的引导作用，促进粮食生产稳定发展。加强对粮食生产、消费、库存及进出口的监测和市场调控，建立和完善粮食安全预警系统和信息发布制度，完善国家粮食储备吞吐轮换机制，科学运用储备、进出口等调控手段，确保粮食供求基本平衡，维护国内粮食市场稳定。
　　加强粮棉等大宗农产品进出口管理。充分利用WTO有关规则，进一步完善粮棉等大宗农产品进出口贸易管理方式，加强和完善进出口配额管理制度。抓紧建立粮食进出口报告制度和监测预警系统，灵活运用进出口手段，为粮棉等大宗农产品总量平衡和调控工作服务，保护农业和农民利益。
　　加强农业生产资料的生产和管理。积极发展农用工业，适时推进化肥价格市场化改革。加强对农资市场的监管，加快推进农资流通领域的市场化改革，建立农资市场信用体系，打击制造和贩卖假冒伪劣商品的行为。
　　(六)增强应对农村重大突发事件的应急反应能力
　　适应形势发展需要，提高对自然灾害、动物疫情、农作物病虫害、外来有害生物入侵、农业转基因生物安全、森林和草原火灾、沙尘暴灾害、农业环境污染、农产品质量安全、农村安全生产事故、假劣农资坑农害农、农产品国际贸易纠纷等重大突发事件的应急反应能力，制定应急预案，建立预警机制。建立农村重大突发事件信息报告制度、发布制度和应急救援物资储备制度，及时、准确、客观、全面地报告和发布有关信息，建立健全应急物资生产、储备、调拨及紧急配送体系，及时对突发事件进行应急处置，最大限度地减少农村重大突发事件造成的损害。
　　(七)保障农村社会事业健康发展
　　努力增加农村社会事业投入。调整财政支出结构，扩大公共财政覆盖农村的范围，认真落实各级政府要将新增教育、卫生、文化等事业经费主要用于农村的战略决策，努力形成稳定的社会事业建设投入保障机制，加快改变农村社会事业落后的状况。
　　创新农村社会事业管理体制和运行机制。完善农村社会事业管理体制，重点巩固“以县为主”的农村义务教育管理体制，强化县级政府对本地区农村义务教育工作的统筹管理。加快落实乡镇卫生院的人员、业务、经费等划归县级管理。鼓励社会力量在政策允许的范围内，以多种形式兴办农村社会事业，扩大筹资渠道，形成以公有制为主体、多种所有制共同发展的格局。推进农村社会事业单位分类管理，按照营利性和非营利性，实行不同的经济政策、管理和运行模式。加强农民自主参与，以满足农民需求为出发点和落脚点，把农村社会事业发展与农村群众的切身利益紧密结合起来，尊重农民意愿和首创精神，在政府支持、引导下，发挥村民自治组织作用，充分激发农民在公共服务决策、投入、管理、监督中的主动性和自觉性。
　　加强统筹规划，按照因地制宜、分类指导、分步实施的方针，把农村社会事业纳入到新农村建设的总体任务中，以规划为先导，整合资源，突出重点，兼顾当前与长远、需要与可能，统筹发展农村社会事业。
　　(八)加强农村法制建设
　　认真执行《[农业法](https://www.pkulaw.com/chl/29b83e1ef6f493c7bdfb.html)》、《[畜牧法](https://www.pkulaw.com/chl/10c83f09be2ffe29bdfb.html)》、《[渔业法](https://www.pkulaw.com/chl/a9844f654f12a37fbdfb.html)》、《[种子法](https://www.pkulaw.com/chl/ccee26d863faa582bdfb.html)》、《[农产品质量安全法](https://www.pkulaw.com/chl/b6f5f01601279950bdfb.html)》、《[动物防疫法](https://www.pkulaw.com/chl/0b4c46aa5685a1cebdfb.html)》、《[农村土地承包法](https://www.pkulaw.com/chl/8ba2c37b78b02a7fbdfb.html)》、《[土地管理法](https://www.pkulaw.com/chl/098c17d5cfc8b2a7bdfb.html)》、《水法》、《[森林法](https://www.pkulaw.com/chl/afdd6dbfcd1389c6bdfb.html)》、《[防沙治沙法](https://www.pkulaw.com/chl/b6e2205864302747bdfb.html)》、《[草原法](https://www.pkulaw.com/chl/9549c12b1ed25195bdfb.html)》、《[农业机械化促进法](https://www.pkulaw.com/chl/88d35d198a08e7cabdfb.html)》、《[气象法](https://www.pkulaw.com/chl/3d46115ef4058b87bdfb.html)》、《[可再生能源法](https://www.pkulaw.com/chl/832c894257ae2b43bdfb.html)》、《[义务教育法](https://www.pkulaw.com/chl/dd44935f74ce51f0bdfb.html)》、《[传染病防治法](https://www.pkulaw.com/chl/7e2d6f8dd0a23fb0bdfb.html)》、《[人口与计划生育法](https://www.pkulaw.com/chl/252f7ce51132230bbdfb.html)》等法律法规，加快有关法律法规的立法进程，依法加强农业基础地位，加快农村经济社会发展，保护农村资源环境，保障农民合法权益。适应新阶段农村经济社会发展和改革的需要，通过法律法规，把近年来行之有效的支农扶农政策措施制度化、规范化。
　　健全农村执法体系，推进农业综合执法，加强执法检查，提高执法水平，为农业和农村经济社会发展保驾护航。深入开展农村普法宣传教育，培养农民法制意识，增强法制观念，提高农民遵纪守法和正确运用法律手段维护自身权益的能力。
　　各有关部门要增强使命感和责任感，按照规划的总体部署，结合本地发展实际，坚持把发展现代农业，繁荣农村经济，增加农民收入，推进社会主义新农村建设，构建农村和谐社会作为工作的重中之重，科学安排政府投资，合理引导社会资源，切实加强对农业和农村经济运行情况的宏观调控，确保顺利实现农村经济社会发展“十一五”规划目标。

© 北大法宝：（[www.pkulaw.com](https://www.pkulaw.com/)) 专业提供法律信息、法学知识和法律软件领域各类解决方案。北大法宝为您提供丰富的参考资料，正式引用法规条文时请与标准文本核对。
欢迎查看所有[产品和服务](https://www.pkulaw.com/helps/2.html)。法宝快讯：[如何快速找到您需要的检索结果？](https://www.pkulaw.com/helps/70.html)[法宝](https://www.pkulaw.com/helps/69.html)V6有何新特色？

扫描二维码阅读原文

**原文链接：**<https://www.pkulaw.com/chl/71a8c2da7db9a28dbdfb.html>
